# Supplementary material for: Identification of photoperiod-induced specific miRNAs in the adrenal glands of Sunite sheep (Ovis aries)
Source: Front Vet Sci. 2022 Jul 22;9:888207. doi: 10.3389/fvets.2022.888207 (PMC9354845; doi:10.3389/fvets.2022.888207)
Supplement: Supplementary file 1 [file Data_Sheet_1.ZIP › Supplementary file/miRDeep2 file.zip!/miRDeep2 file/Know_Structure/SP42A3/oar-mir-26a.pdf]

miRBase precursor : oar-mir-26a
Total read count : 1314886
oar-miR-26a read count : 1314793
remaining reads : 93

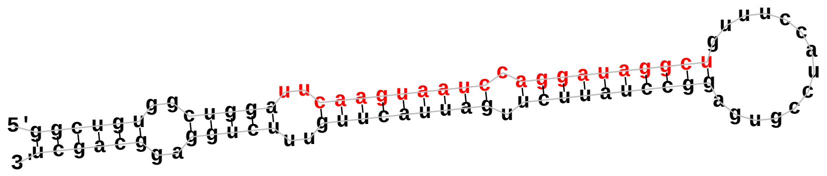

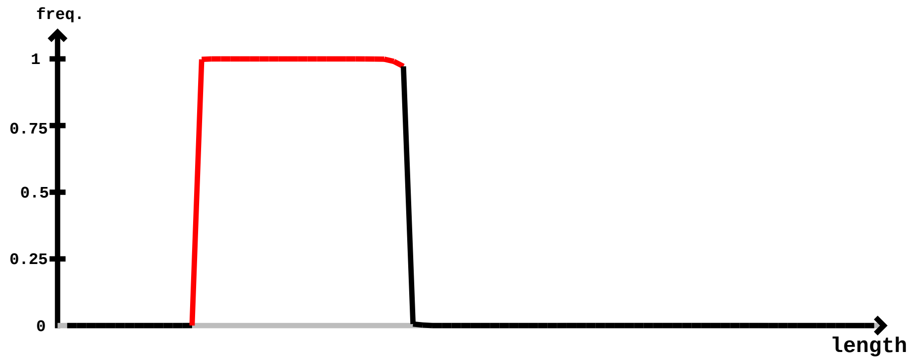


```
                 oar-miR-26a
5'- ggcuguggcuggauucaaguaauccaggauaggcuguuuccauccgugaggccuauucuugauuacuuguuucuggaggcagcu  -3'    exp
    ((((((..(((((..(((((((((.((((((((((..............)))))))))).)))))))))..)))))..))))))    reads      mm         sample
    ...........auucaaguaauccaggauag.....................................................    2          0          seq
    ...........auucaaguaauccaggauagg....................................................    4          0          seq
    ...........Guucaaguaauccaggauaggc...................................................    3          1          seq
    ...........auucaaguaauccaggauaggc...................................................    9          0          seq
    ...........auucaaguaauccaggauaggcu..................................................    238        0          seq
    ...........Guucaaguaauccaggauaggcu..................................................    32         1          seq
    ...........auucaaguaauccaggauaggcug.................................................    2          0          seq
    ............uAcaaguaauccaggau.......................................................    2          1          seq
    ............uucaagGaauccaggau.......................................................    1          1          seq
    ............uucaaAuaauccaggau.......................................................    1          1          seq
    ............uucaaguaauccaggCu.......................................................    1          1          seq
    ............uucaGguaauccaggau.......................................................    2          1          seq
    ............uucaaguaauccaggaG.......................................................    8          1          seq
    ............uuAaaguaauccaggau.......................................................    1          1          seq
    ............uucaaguaauccaggau.......................................................    312        0          seq
    ............uucaaguaauccaggaA.......................................................    9          1          seq
    ............uucaUguaauccaggau.......................................................    1          1          seq
    ............uucaaguaauccaggaua......................................................    415        0          seq
    ............uucaaAuaauccaggaua......................................................    1          1          seq
    ............uucaGguaauccaggaua......................................................    6          1          seq
    ............uucaaguaauccaggauag.....................................................    545        0          seq
    ............uucaaguaauccaggauagg....................................................    10961      0          seq
    ............uucaaguaauccaggauaggc...................................................    24396      0          seq
    ............uucaaguaauccaggauaggcu..................................................    1267930    0          seq
    ............uucaaguaauccaggauaggcug.................................................    4964       0          seq
    ............uucaaguaauccaggauaggcugu................................................    2565       0          seq
    ............uucaaguaauccaggauaggcuguG...............................................    2          1          seq
    ............uucaaguaauccaggauaggcuguu...............................................    35         0          seq
    ............uucaaguaauccaggauaggcuguuu..............................................    6          0          seq
    ............uucaaguaauccaggauaggcuguuuc.............................................    2          0          seq
    ............uucaaguaauccaggauaggcuguuuccauc.........................................    1          0          seq
    .............ucaaguaauccaggauag.....................................................    1          0          seq
    .............ucaaguaauccaggauagg....................................................    13         0          seq
    .............ucaaguaauccaggauaggc...................................................    41         0          seq
    .............ucaaguaauccaggauaggcu..................................................    2136       0          seq
```

oar-miR-26a

```
ggcuguggcuggauucaaguaauccaggauaggcuguuuccauccgugaggccuauucuugauuacuuguuucuggaggcagcu
..............ucaaguaauccaggauaggcug................................................   21   0   seq
..............ucaaguaauccaggauaggcugu...............................................   13   0   seq
...............caaguaauccaggauaggc..................................................   4    0   seq
...............caaguaauccaggauaggcu.................................................   91   0   seq
...............caaguaauccaggauaggcug................................................   1    0   seq
................aaguaauccaggauaggcu.................................................   16   0   seq
......................................uccauccgugaggccuauucuug.......................   1    0   seq
................................................aggccuauucuugauuacuugu..............   1    0   seq
................................................aggccuauucuugauuacuuguu.............   3    0   seq
................................................aggccuauucuugauuacuuguuu............   2    0   seq
...................................................ccuauucuugauuacuuguu.............   5    0   seq
...................................................ccuauucuugauuacuuguuu............   37   0   seq
...................................................ccuauucuugauuacuuguuuc...........   41   0   seq
....................................................cuauucuugauuacuuguuu............   1    0   seq
....................................................cuauucuugauuacuuguuucu..........   1    0   seq
```
